# Well-Being and Cooking Behavior: Using the Positive Emotion, Engagement, Relationships, Meaning, and Accomplishment (PERMA) Model as a Theoretical Framework

**DOI:** 10.3389/fpsyg.2021.560578

**Published:** 2021-04-12

**Authors:** Nicole Farmer, Elizabeth W. Cotter

**Affiliations:** ^1^National Institutes of Health, Clinical Center, Bethesda, MD, United States; ^2^Department of Health Studies, American University, Washington, DC, United States

**Keywords:** well-being, positive psychology, cooking, PERMA, theory

## Abstract

The prevalence of psychosocial distress is increasing in the United States. At the same time, the American default lifestyle has steadily displaced household food production with industrial food production, despite increased cultural interest in cooking. An important focus of cooking research to date has been on cooking’s association with nutrition and dietary quality. Less focus has been placed on how cooking might foster the qualities that allow for mitigation of psychosocial distress and promote well-being. Rooted in its evolutionary role in the human experience, cooking requires skills and knowledge that have the capacity to encourage aspects of well-being as described by Seligman as flourishing. Evidence for a beneficial role of cooking in psychosocial health exists, but the exploration is limited, potentially due to lack of a theoretical context to explain these benefits. From this perspective, we review the current literature showing the application of Seligman’s prominent well-being model, Positive emotion, Engagement, Relationships, Meaning, and Accomplishment (PERMA), to cooking, defined as the activity related to the preparation of food or a meal. We propose that the PERMA model as applied to cooking may function as a theoretical framework to explore psychosocial outcomes associated with cooking. Broader application of this approach may also help to further the application of positive psychology in the developing literature around psychosocial health and nutrition-related chronic diseases.

## Introduction

Recent attention has been given to the role of psychosocial health and well-being in the etiology of various health outcomes including cardiovascular disease and mental health conditions (Everson-Rose and Lewis, 2005; Whang et al., 2009; Mueser et al., 2013). The importance of well-being to individuals and overall society has captured the interest of public health organizations in recent years as they seek to measure and identify ways to increase well-being (Kottke et al., 2016). Despite this interest, life satisfaction remains unchanged (Diener and Seligman, 2004), and rates of depression and anxiety have increased in the United States despite economic growth over the last 5 decades (Robins et al., 1984; Klerman et al., 1985; Hidaka, 2012; Twenge et al., 2019). The current modern American lifestyle, which consists of displacing human energy with mechanical energy and displacing household food production with industrial food production, may contribute negatively to our psychosocial health. Reduced use of our hands (Lambert, 2006) for production and less attention required to participate in lifestyle activities may contribute to psychosocial decline. Based on this premise, the increase in psychosocial distress in the United States in recent years (Kessler et al., 2005) may represent a confluence of issues related to daily lifestyle. One way to help mitigate the American default lifestyle on psychosocial distress may be to leverage opportunities within daily life to engage in healthful behaviors. We hypothesize that cooking is such a behavior.

Positive psychology is “the scientific study of the strengths that enable individuals and communities to thrive” or flourish (VanderWeele, 2017, p.1). Identifying daily behaviors, which contribute to or are associated with these strengths is important. Applying the Positive emotion, Engagement, Relationships, Meaning, and Achievement (PERMA) model to health behaviors can increase understanding of their contributions to well-being and flourishing. Cooking may be a candidate behavior. Within this argument is the understanding that if cooking is related to positive psychology, then it must be determined by and have benefits related to elements of well-being. Similarly, there has been burgeoning interest in the role of diet in promoting well-being. Consumption of fruit and vegetables is linked to more positive well-being (Blanchflower et al., 2013; Mujcic and Oswald, 2016) but despite the fact that fruit and vegetable consumption is associated with cooking (Soliah et al., 2012), there is scant evaluation of the role of cooking in promoting well-being. This omission of cooking is perhaps reflective of the general perception of cooking as a chore-based activity and not an opportunity for improved well-being.

**Figure 1:**
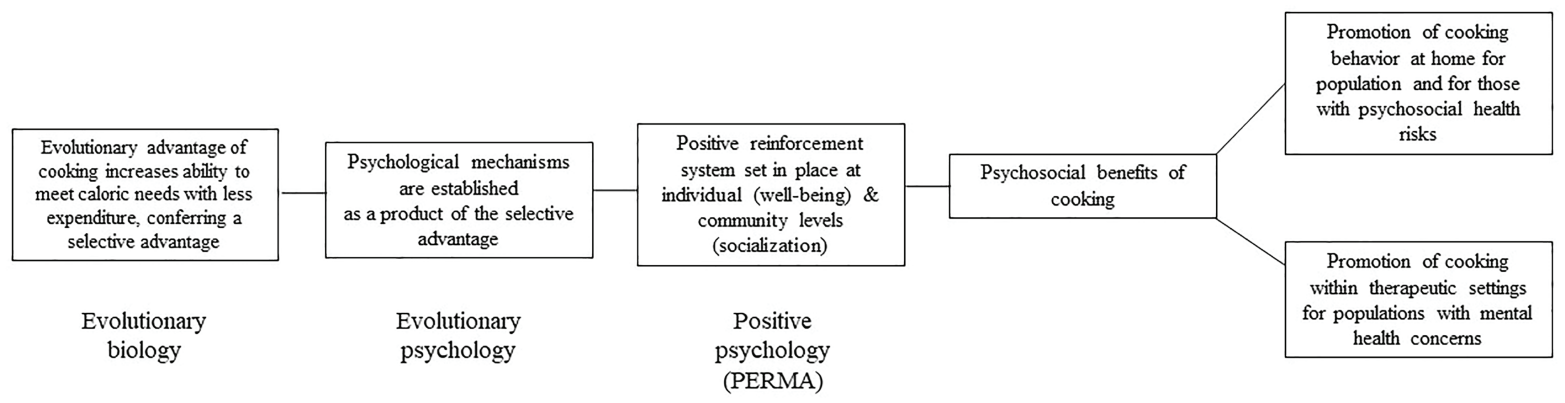
Concept model of psychosocial benefits of cooking showing multi-theoretical basis and proposed applications for overall health, including both mental and physical health.

Evaluation of cooking through an evolutionary, archeological, and anthropological premise may provide a basis for our argument to evaluate not only “what we eat” for well-being but also “what we do to eat” as connected to our well-being. However, even taking this point of view may not tell us what processes ensure people will engage in cooking. A framework for identifying these processes is still needed to drive the generation of hypotheses and to start empirical evaluation. The following perspective article seeks to provide an initial exploration into potential hypotheses linking cooking and well-being through the perspective that cooking is an evolutionarily essential behavior for the establishment of psychosocial health (Figure 1). We also explore well-being as a premise to explain the psychosocial benefits of cooking and discuss potential areas of investigation within the field of cooking behavior that merge principles of positive psychology and behavioral medicine to put forth recommendations for incorporation of these principles into future cooking research and interventions.

### Introduction to Cooking Behavior

We suggest that cooking is a health behavior that represents human flourishing, or in the least the capacity to flourish. Archeological studies conducted by Wadley et al. (2020) show the role that cooking of tubers had on human civilizations and the capacity to expand out of Africa 170,000 years ago. While evolutionary biology studies have identified the role of cooking on meeting caloric needs required for human cortical development, specifically the prefrontal cortex (Aiello and Wheeler, 1995), anthropological evidence exists for the role of cooking in socialization and sharing of customs and celebrations among societies (Wrangham et al., 1999). Indeed, recent qualitative research with adults in the United Kingdom and the United States suggests that a range of social and emotional benefits stem from home cooking (Mills et al., 2020), indicating that future research should examine these benefits rather than solely cooking’s influence on physical health.

Cross-sectional and short term intervention research demonstrate associations of cooking with vegetable intake, lower daily calories, and reduced empty calorie consumption; dietary characteristics that fall in line with the current WHO’s Global Action Plan for the Prevention and Control of Non-communicable Diseases (World Health Organization, 2013). To date, outcomes from cooking interventions, along with association of cooking frequency among the United States population have not yet shown consistent evidence of improvement in relevant physical health outcomes (Reicks et al., 2014, 2018; Hasan et al., 2019). Although not in opposition to a nutrition-oriented approach toward cooking research, better understanding of the intersection between cooking and well-being may yield interventions that can help further promote cooking’s role in mitigating adverse outcomes such as high body mass index and poor dietary quality. Appreciation of cooking from this standpoint requires a view in which the behavior that leads to a consumable product is just as vital as the product created. In other words, if the consumed product can contribute to wellness, then so can the behavior to create the product. Although not widely held within Western cultures, the notion of not splitting the psychosocial benefit of the consumable product from the process itself is evident in anthropological studies from other cultures (Kubovy, 1999).

Cooking involves simultaneous and mixed use of multiple skills. Specifically described by Short (2003), skills involved in cooking may be categorized as: cognitive, planning, perceptual, and mechanical. Cooking skills may explain the relationship between well-being and cooking in two ways. First, the use of cooking skills creates the potential for neurobiological activation. For example, mechanical cooking skills are underpinned by fine and gross motor skills (Dean et al., 2021). This movement may interact with neurobiological pathways such as the serotonergic or dopaminergic pathways (Lambert, 2006). Cooking may then mitigate psychological distress (stress, depression, and anxiety) as evidenced by reports of psychosocial benefit to cooking (Hill et al., 2007; Farmer et al., 2018). Second, skills involved in cooking are representative of life skills, such as problem-solving and planning, which are identified by the World Health Organization (2013) as fundamental to reaching psychosocial competence.

### Modern Perceptions of Cooking

Most United States households report cooking dinner the majority of nights per week (Wolfson and Bleich, 2015), with cooking frequency for some, but not all (Farmer et al., 2019) demographic populations in the United States increasing from 2003–2016 (Taillie, 2018). Time-use surveys indicate that adults often view cooking as a household chore rather than a meaningful activity, although this depends on context (Daniels et al., 2012). Common barriers to cooking include time and money constraints, lack of energy to cook, past cooking failures, perceived lack of enjoyment, and tiredness (Gatley et al., 2014; Lavelle et al., 2016; Wolfson et al., 2016; Mills et al., 2017). An activity that is viewed from a chore perspective, or one that is less interesting, requires more of this directed attention, and mental or attention fatigue may occur (Kaplan, 2001). Although not studied directly, it is reasonable to conclude that anticipation of this type of mental fatigue could prevent an already tired person from engaging in cooking and create attitudes of less enthusiasm (e.g., the thought of cooking after a long workday). Indeed, the average total amount of time spent cooking has decreased to less than 20 min per meal (Taillie, 2018).

Reports of barriers to engaging in cooking may lead one to conclude that cooking is a threat to well-being. There is evidence that the removal of certain cooking barriers (e.g., lack of time) increases adults’ involvement in dinner preparation from scratch (Murphy et al., 2021). Moreover, there is a current cultural attraction to cooking as noted by media attention to cooking shows (Worsley et al., 2014). This affinity for cooking mirrors the contemporaneous popularity of craft consumerism (i.e., “crafting”; Campbell, 2005) and thus may reflect, at least for the middle class, the resources and ability to utilize cooking as an outlet for expression and autonomy in the face of food commodification and modernity. Both cultural trends may be insinuations of innate attractions to the use of hands and creating. Cooking interventions also consistently show an increase in cooking self-efficacy and cooking confidence (Crookes et al., 2016; Mills et al., 2017). These cooking-related psychologically beneficial outcomes might be representative of an underappreciated connection of cooking with psychological well-being and more global psychosocial benefits. But with the exception of a few studies (Herbert et al., 2014; Barak-Nahum et al., 2016; Mills et al., 2020), psychosocial outcomes in cooking research remain under-investigated. This discrepancy in the cooking literature may be a function of a lack of a behavioral science perspective in cooking interventions, along with a lack of a theoretical model to apply. Integration of a positive psychology framework, such as PERMA, might fill this gap by addressing motivation for health behavior engagement that leads to flourishing.

### Introduction of the PERMA Model

Currently there is a growing belief that psychology research can and should be used not only to identify pathological psychological conditions but also to identity and promote adaptive psychological states as well. The promotion of well-being is a manifestation of this growing belief. Well-being is a multi-faceted construct that includes emotional, social, and functional components (Diener and Ryan, 2009). Broad definitions of well-being include happiness, quality of life, life satisfaction, and flourishing. Although originally focused on the concept of happiness, Seligman (2011, p. 97) later provided a paradigm shift through a broader focus on well-being in the field of positive psychology in order to capture “what humans pursue for their own sake.” PERMA provides a model of the proposed building blocks of well-being (Seligman, 2018). Work by Goodman et al. (2017) has shown that PERMA is in fact representative of subjective well-being, and that the presence of one component in a person means that other components are likely to be present.

One of the unique aspects of the PERMA model, which distinguishes it from other frameworks of well-being is the inherent connection to eudemonic well-being (i.e., well-being stemming from personal fulfillment or meaning), and the focus on application of the PERMA domains within one’s life. As such, PERMA offers a multi-dimensional operationalized definition of well-being that helps someone involved in an activity move beyond a success/fail evaluation; to one that evaluates relative strengths and weaknesses of an activity’s effects on the different key well-being dimensions (Friedrich and Mason, 2018). The PERMA model has been applied to interventions intended to reduce depressive symptoms (Gander et al., 2016) and burnout (Slater et al., 2018) and to improve the aging process (Bartholomaeus et al., 2019). Studies have also applied PERMA to daily activities, such as physical activity and music (Lee et al., 2017; Ascenso et al., 2018). Similar to cooking, physical activity can be viewed as a lifestyle-based activity with an evolutionary advantage for survival. Mutrie and Faulkner’s (2004) work on physical activity and well-being stresses the importance of understanding and evaluating the *process of the activity* for an individual – an approach that we will take in our evaluation of cooking and well-being. Within the context of PERMA and music, attention has been paid to not only music’s potential value, but also its potential to be a source of anxiety and stress that could manifest as a triad of cognitive, somatic, and behavioral symptoms (Craske and Craig, 1984). Yet, Ascenso et al. (2018) have shown the importance of applying the PERMA model to those who create music in order to create a well-being profile. Their work demonstrates the idea that a re-direction toward positive assessment of well-being and related theory-driven research may uncover new appreciations for activity participation thought to be tedious or adverse to well-being. We therefore evaluated peer-reviewed and published studies of cooking activity and behavior as well as studies from the positive psychology literature to try to uncover new appreciations between these fields.

## Perma Model and Cooking

### Positive Emotion

According to Seligman’s PERMA model (2012), positive emotion extends beyond happiness and includes feelings of gratitude, love, optimism, and contentment. Positive emotion is momentary and can vary distinctly throughout the day, depending on a person’s location and the activities they are engaged in (Stone et al., 1996). Positive emotion may also drive our thoughts and actions, promote resilience, and undo negative emotions (Fredrickson et al., 2003). Experiences of positive emotion may prompt individuals to engage with their environments and partake in activities that are adaptive for the individual, its species, or both (Fredrickson, 2001). The role of positive emotion within cooking may also complement our hypothesis that participation in cooking is a part of a feedback reward system to ensure ongoing participation in the behavior. It is well-recognized that, from an evolutionary standpoint, pleasurable reactions help to shape behavior (Berridge and Kringelbach, 2013). The clinical importance of identifying the role of positive emotion in our understanding of the relationship between cooking and health is highlighted in studies that link positive affective states to favorable profiles of physical health, including more favorable levels of cortisol and plasma fibrinogen (Steptoe et al., 2005). Therefore, identifying opportunities for positive emotion to occur within cooking may be the missing link needed to help investigate changes in physical health from cooking, since changes in dietary intake have not done so thus far (Reicks et al., 2014; Hasan et al., 2019).

Although few cooking intervention studies have examined cooking’s influence on emotions; several recent studies suggest that cooking interventions may be predictive of increased positive affect (Jyväkorpi et al., 2014; Adam et al., 2015; Barak-Nahum et al., 2016). For instance, a three-session nutrition education and cooking class intervention for older adults yielded improvements in self-reported psychological well-being post-program (Jyväkorpi et al., 2014). Participation in a 5-week online nutrition and cooking education course geared toward parents from over 80 countries was associated with greater reports of enjoyment during meal preparation from baseline to post-program (Adam et al., 2015). Perhaps then it is no surprise that one of the most popular and best-selling cookbooks of all time is titled, “The Joy of Cooking” (Rombauer et al., 2019). Interest creates the urge to explore, take in new experiences and expand the self (Csikszentmihalyi, 1990). The positive emotion of pride promotes sharing the news of achievement with others and helps to envision future achievements (Fredrickson, 2001). Both interest and pride in relation to cooking will be further explored in the PERMA domains of engagement and achievement, respectively. Conversely, cooking does not always lead to an onset of positive emotion as explored in a qualitative study by Lavelle et al. (2016) of adults from Ireland in which making scratch meals was described as leading to feelings of inadequacy and fear of provoking family and self-disappointment. This provides support for strategies that can promote the development of new cooking skills and techniques that might enhance positive emotions associated with cooking.

### Engagement

Seligman (2011) states engagement is about flow, or being one with an activity, experiencing a sense of time stopping, and the loss of self-consciousness during an activity. Flow experiences involve being attentively absorbed and skillfully engaged in an activity that one finds valuable (Csikszentmihalyi, 1990). Kubovy (1999) separated Csikszentmihalyi’s characteristics of a flow activity into distinct categories: (1) the nature of the activity is challenging but doable with defined goals and immediate feedback available to the participant; (2) the effect of the activity allows the participant to feel in control; (3) the nature of involvement in the activity leads to immersion of self; and (4) the effects of the activity makes the participant forget themselves and time slows down. Ultimately, reaching the flow experience in an activity is relevant to well-being as it can invoke a sense of virtuosity as a result of performing at a high level.

Similar to other food-related behaviors, such as eating and gardening (Soga et al., 2016; Holder, 2019), opportunities for engagement during the cooking process are multiple. Regarding cooking skills, there is promotion of a mixed-use of abilities that include motor coordination, thinking, flexibility and implementing strategies – all abilities that may translate into characteristics that are related to psychosocial health. How people reach engagement in cooking, maintain it, and then also benefit from the engagement is important to determine. Large, cross-sectional studies with adults suggest that alterations in foodwork, such as batch cooking or pre-chopping, are utilized to help maintain enthusiasm to cook (Mills et al., 2017). A further explanation for the potential for engagement in cooking might be through exploring attention restoration theory (ART; Kaplan, 2001), which involves the use of a person’s stated enjoyment or involvement in an activity to limit the capability of directed attention to create fatigue and concentration difficulties. Notably, ART is reported as a way to also introduce pathways for stress reduction (Kaplan). There are four stated qualities of an attention restorative activity that overlap with flow: (1) distinctiveness from the everyday environment; (2) fascination to hold attention effortlessly; (3) presence of scope and coherence to allow one to remain engaged; and (4) compatibility with what one is inclined to do. Each of these might occur during the cooking process. Distinction may occur when cooking is considered as a break, time to one’s self, or time for social enjoyment as described by Irish mothers in semi-structured interviews (Lavelle et al., 2016). Scope and coherence relate to the perceived depth that one has regarding ability and being in an environment that is supportive of the activity. For example, a coherent environment according to Kaplan is one in which things follow each other in a sensible, predictable, and orderly way. Within the kitchen, this may occur through the placement of supplies and equipment in easily useable fashion as measured by occupational therapy standardized cooking task assessments for process skills (Birnboim, 2001) or through the culinary use of “mis-en-place” to prepare and arrange ingredients of a recipe (Wolfson et al., 2017). Despite these correlates with cooking, the presence of attention restoration has not been evaluated within cooking tasks.

### Relationships

Positive relationships and social connections are central to health and well-being (Ryff and Singer, 2001; Cohen, 2004). Individuals with adequate naturally occurring social relationships have a 50% greater likelihood of survival over a 7.5 year period compared to those with poor or insufficient social relationships, and this effect is present after controlling for demographic and health status factors (Holt-Lunstad et al., 2010). The effect of social relationships on longevity is comparable to well-established risk factors for mortality, such as quitting smoking, obesity, and physical inactivity. Interestingly, social integration is more strongly associated with survival than received support (i.e., support that comes from hired personnel; Holt-Lunstad et al., 2010). Thus, promoting naturally occurring social relations may have more of an overall health effect. Work initiated by Cohen et al. (1997) and supported by others demonstrates the role of social relationships on physiologic processes (Uchino et al., 1999), including immune mediated inflammatory responses (Moynihan et al., 2004) and epigenetic effects (Meloni, 2014; Cunliffe, 2016).

Anthropological research suggests that humans have been preparing and sharing cooked meals together for centuries (Wadley et al., 2020). It is hypothesized that some of the pleasure derived from cooking stems from food’s positive associations with social occasions and time spent with loved ones (Bublitz et al., 2013). Improved well-being from social relationships may be explained by the buffering hypothesis, which states that receiving meaningful social support can protect people from experiencing negative effects of stress (Whillans et al., 2017). Social relationships also may act as mediators for engagement in better health practices that increase subjective well-being. Despite these clear positive effects, challenges to effectively utilizing the benefits of social relationships abound, especially in the face of modern life. Behavioral scientists Epley and Schroeder (2014) suggest that we consider moments embedded within modern life as opportunities for connecting with others. Qualitative research with adults highlights that a central benefit of home cooking is that it facilitates this social connection (Simmons and Chapman, 2012). Qualitative research with older women similarly indicates that the meaning in cooking is found through preparing a “gift” for others to enjoy (Sidenvall et al., 2000). Regarding parent–child relationships, direct observational research with families suggests that the cooking and serving of family meals provides an important opportunity for parents to role model healthful eating habits and to communicate positively about food (Berge et al., 2014). Indeed, opportunities to foster relationships occur across the spectrum of cooking, from thinking about and discussing others’ preferences when meal planning, to teaching others how to cook, to enjoying a meal with others, to continuing family and cultural culinary traditions. At present, however, there is limited evidence that cooking consistently promotes socialization within the family and little is known regarding the centrality of actual cooking, vs. the sharing of a meal more generally (i.e., a meal prepared by others, takeout), in fostering social connections (Short, 2006).

### Meaning

According to Seligman’s (2011) PERMA model, meaning refers to one’s belief that their life is valuable and that they are connected to something greater than the self. Qualitative interviews with adults highlight the meaning found in cooking through the sense of fulfilling a role or responsibility, such as the role of a parent, spouse, or caregiver (Mills et al., 2020). Adults completing daily time-use surveys describe cooking as more meaningful when there is not a sense of time pressure, when meals are cooked with others, when food is being prepared for others, and when more time is spent on preparation (Daniels et al., 2012). In further qualitative studies with parents and their teenagers (Simmons and Chapman, 2012), parents reported that cooking gave them a sense of control in their role as guardians of their children’s health (e.g., the ability to screen unhealthy ingredients). Teenagers described cooking as an opportunity to gain responsibility and become more independent. As stated above in the Relationships section, participants in qualitative research describe cooking as also providing meaning through the opportunity to connect and share with others (Sidenvall et al., 2000; Simmons and Chapman, 2012). Perhaps the meaning found in cooking as already been highlighted in the field of occupational therapy, which is focused on increasing people’s ability to develop and maintain meaningful activities (Duncan, 2004, p. 198). Occupational therapy scholars suggest that regular engagement in purposeful and meaningful activities (such as cooking) facilitates healthy emotional development.

### Achievement

Achievement (i.e., accomplishment) may occur through production of a tangible object or through demonstration of skill or mastery. The well-documented “IKEA effect” indicates that people value their own creations more than objectively similar products created by others (Marsh et al., 2018). Making of a product is an inevitable final component of cooking and extant research demonstrates that people prefer self-prepared meals to meals prepared by others (Dohle et al., 2014). This type of end result may lead to not only happiness-based well-being but also a eudemonic one focused on a sense of purpose or personal growth. A key driver of achievement is self-efficacy, which refers to one’s belief about their capability to perform successfully in a certain domain (Bandura, 2010). An efficacious outlook fosters intrinsic interest in a task, engagement, and well-being (Bandura, 2010). A wealth of studies have linked cooking interventions to increases in cooking self-efficacy and confidence in one’s cooking ability from baseline to post-intervention (e.g., Levy and Auld, 2004; Hersch et al., 2014; Garcia et al., 2016). Although expected as a result of participation in interventions designed to increase skills, the role of ability and mastery have also been found in non-intervention studies. A survey of 8,500 adolescent students found that greater confidence in cooking abilities was linked to lower levels of depression and higher levels of well-being (Utter et al., 2016). Sense of achievement, while cooking aligns with the social cognitive theory (Bandura, 2005), which describes people as agents who intentionally influence their functioning and life circumstances. From this perspective, a home cooked meal then provides an opportunity for personal accomplishment and improved life functioning, as has been described in qualitative research with women and families (Moisio et al., 2004; McCabe and de Waal Malefyt, 2015). Interestingly, this may link achievement to the domain of positive emotion. One such positive emotion, virtuosity, represents the pleasure that occurs when a person feels they are doing something well (Kubovy, 1999). It does not require the achievement to be extraordinary. It requires that a person perform or do something that they once could not do before, and thus the experience of virtuosity is in relation to a previous lack of ability (Kubovy, 1999).

Development of comprehensive skill in a task occurs with repeated exposures and experiences. Use of tacit skills is associated with reported enjoyment from cooking (Short, 2006). This aligns with research indicating that experiential cooking classes outperform cooking demonstrations in improving attitudes toward cooking (Levy and Auld, 2004), likely because of the opportunity to use tacit skills and gain mastery in cooking. Thus, identifying ways to allow opportunities for achievement, such as more opportunities for hands-on cooking experiences, might be beneficial from a well-being perspective. It is unknown if current trends in the use of convenience foods or reduction in time spent cooking as a function of altered foodwork is helpful or hurtful to the attainment of mastery. Some have argued that it is disruptive in the attainment of skills (Lang and Caraher, 2001); however, work presented by Short (2007) from the direct observation of families cooking posits that there is maintenance of skill transference, which might contribute to family well-being when cooking with convenience foods.

### Intersections Between PERMA Domains Within Cooking

#### Present Moment Awareness

Although the PERMA model was constructed on the basis of distinct domains, overlap and intersection between the domains has been reported by Goodman et al. (2017). Present moment awareness, or mindfulness, within cooking may represent such overlap. Just as mindfulness can be cultivated in daily activities such as walking and breathing, we argue that cooking provides an opportunity for focus and present moment awareness. Humans are noted to engage in mind wandering, or the regular entertainment of thoughts not relevant to the current moment (Langer, 1992). This was possibly an evolutionary advantage designed to allow people to learn, reason, and plan; however, it comes with the emotional cost of greater unhappiness (Killingsworth and Gilbert, 2010). As mentioned earlier, there is a significant role for planning within cooking. This planning is in fact controlled thoughts, and not stimulus independent thought, with regard to next steps, reasoning and learning needed to complete a cooking task. In this, the planning occurring within cooking is quite different from the planning associated with mind-wandering. Theoretically, greater present moment awareness increases positive emotion by enhancing one’s awareness of the positive aspects of an experience and the associated positive emotions (Brown and Ryan, 2003; Tugade and Fredrickson, 2007). Present moment awareness is also linked to enhanced creativity (Greenberg et al., 2012). Cooking offers numerous opportunities to express oneself creatively according to qualitative research, including exploring new foods and flavor profiles; examining how ingredients work together; successfully preparing meals despite time, money, or family preference constraints; creating dishes that are visually pleasing or colorful, and recreating meals seen outside the home (Short, 2003; Gatley et al., 2014; McCabe and de Waal Malefyt, 2015). Despite the potential connection between cooking and present moment awareness, there currently is limited empirical evidence regarding this connection in the literature.

#### Time Perception

Although fewer Americans today report not having sufficient time to get everything they need to do done compared to past decades (Saad, 2017), stress levels have not decreased. This suggests the use of technology is providing more efficient use of time, but no subsequent relief in stress. Despite the time perception improvement, those who are employed and have children report the greatest time pressures and stress (Saad, 2017). Presumably, use of technology has not alleviated time pressure or perception for this demographic. Psychological research on time perception may provide an approach, coupled with the PERMA understanding of cooking, to potentially increase interest in cooking and its associated benefits, beyond promoting cooking’s nutritional benefits. In particular, research shows that spending time on others (e.g., cooking for others) may promote prosocial behavior and change the perception of time (Grant and Gino, 2010; Gray, 2010). However, if time is felt to be given away for obligatory reasons, or too much time is given so that it compromises the giver’s ability to take care of their own tasks, then giving of time was felt to be wasted time (Mogilner et al., 2012). Thus, there is a dosage limit for a positive effect. In relation to cooking, this could be prevented by promoting task sharing in cooking responsibilities rather than just one person having the obligatory responsibility.

### Discussion

#### Research and Clinical Implications

##### Characterizing Cooking

Although the definition of cooking varies, there are certain commonalities that span various definitions, such as the use of available food resources, application of a method for processing (e.g., changing of food through heat), use of senses, and production of a product for consumption. One’s perception (or individual-level definition) of cooking is important to identify and could impact their association between cooking and well-being. Work done by Wolfson et al. (2016) has shown that cooking confidence and enjoyment are lowest among surveyed Americans who perceive cooking as including the use of convenience foods. While this finding supports the notion that home cooking has relevant elements of the PERMA model, it also showcases the importance of understanding how individuals define cooking before applying the model. If cooking in the real-world encompasses PERMA domains, then cooking interventions should represent this real-world experience in order to demonstrate well-being outcomes. The extent to which relevant PERMA domains may be identified in an intervention should be explored in parallel research within home environments in order to inform how to optimize home cooking for increased enjoyment or confidence in daily life. Ethnographic studies within homes may have a sensitivity effect that alters the home cook’s activities; therefore, use of technology such as the e-button (Raber et al., 2018) coupled with real time assessment of well-being may help to best inform the research field. Researchers could also leverage popular interest in cooking on social media by thematically analyzing social media posts related to cooking and PERMA-related terminology.

##### Identifying Behavioral Mechanisms of Cooking Interventions

Well-being is not simply a lack of negative psychological states; it is defined as something positive and worthy of pursuit (Seligman and Csikszentmihalyi, 2000). Comparably, promotion of cooking is not just the removal of barriers, such as time, but involves identifying what needs to be present for the pursuit of the behavior to occur. Attention to relevant mechanisms of behavior change within cooking research has drawn recent attention to cognitive- and motivation-based theories, such as SCT, to explain behavior in cooking interventions (Hollywood et al., 2018). Mapping of cooking relevant to PERMA domains may help aid further investigations. For example, for certain individuals or settings, achievement may be found as a predominant domain. Subsequently, mastery and self-efficacy-based behavioral frameworks may then become identified as important in promoting cooking. This could be explored through use of factorial intervention designs or behavioral adapted designs. Additionally, the use of the PERMA model as a framework for qualitative cooking research may allow for insight about the social and structural factors that pose as motivators or barriers to cooking.

##### Addition of Positive Psychology to Cooking Interventions

Positive psychology interventions have been defined as “[…] treatment methods or intentional activities that aim to cultivate positive feelings, behaviors, or cognitions” (Sin and Lyubomirsky, 2009, p. 468). Inclusion of exercises based on an aspect of positive psychology, such as gratitude, can be done within interventions framed by the PERMA model to promote cooking. Evidence put forth by Seligman (2010) indicates that the use of positive psychology exercises within interventions leads to self-sustaining behavior post-intervention. Candidates for positive psychology strategies that can be incorporated into cooking might include gratitude (e.g., taking a moment to be thankful for the ability to purchase ingredients or enjoy a meal), sensory awareness (e.g., savoring a meal using the five senses), or self-compassion (e.g., being kind to oneself when a meal does not turn out as planned).

##### Exploring Cooking’s Role in Mental Health

Given the apparent link between cooking, meaning-making, and a more positive sense of self, we believe there is untapped potential to examine the benefits of cooking interventions on mental health outcomes and disorders. To date, the limited literature on cooking and mental health has encompassed interventions at the tertiary level, where the goal is mitigation of symptoms through engagement in an activity that fosters a positive sense of self. Qualitative research with individuals with schizophrenia suggests that participation in meaningful daily activities and experiences promotes a more positive sense of self and decreases chance of hospitalization (Sells et al., 2004). To some extent, this has been seen in the use of behavioral activation to treat depression, a psychological treatment that encourages an individual to increase engagement in positive activities including cooking (Cuijpers et al., 2007). Positive reinforcement can be represented through a sense of accomplishment through action (e.g., cooking), but when positive reinforcement is diminished then the opportunity for depression may occur. We thus conclude that cooking might be an under-researched strategy for potentially providing meaning, purpose, and positive reinforcement to those experiencing mental health concerns through engagement in a fulfilling activity. Future research should examine the potential for cooking to improve mental health through the development of interventions at not only the tertiary, but also the primary and secondary levels of prevention, to reach individuals at heightened risk for or with early indications of mental health concerns.

##### Nutritional Health and Well-Being

Cooking at home is associated with higher diet quality and nutrient intake (Smith et al., 2010; Monsivais et al., 2014; Wolfson and Bleich, 2015). This suggests there may be an additional pathway through which cooking interventions improve emotional well-being – through improved dietary intake, which in turn has positive impact on the brain and well-being. Research supports a consistent, cross-sectional relationship between quality of diet and mental health concerns (Marx et al., 2017). Higher quality diets are associated with improved depression and anxiety symptoms in both cross-sectional and intervention studies (Lai et al., 2013; O’neil et al., 2014), although not all research supports this association and more rigorous trials are needed (Opie et al., 2015). Biological pathways through which improved diet might enhance mental health and well-being include improvements in markers of inflammation, increased intake of antioxidants, which reduces oxidative stress, and changes in the microbiota gut-brain axis (Marx et al., 2017). The use of subjective well-being measurements is also not without critique, although more recent validation and psychometric testing of instruments such as the PERMA profiler may provide valid mechanisms for measurement (Butler and Kern, 2016). Outside of survey responses, researchers might also consider more objective measures of psychosocial well-being, such as cortisol levels, blood pressure, sleep duration, or immune function. Future research should also longitudinally examine how cooking influences eating behaviors over time, along with whether changes in eating behaviors might mediate the association between cooking and objective and subjective measures of well-being.

#### Social Implications

##### Gender and Cooking

Inherent within cooking is a gender differential brought on by societal expectations. Women are often the expected culinary and nutritional caregivers of a household, leading to unique pressure on women to be involved in cooking. These societal and household pressures might blunt the expected well-being benefits associated with cooking, as demonstrated in research suggesting that time spent in obligatory tasks is perceived as less rewarding and more stressful (Saad, 2017; Mogilner et al., 2018). Indeed, historically cooking interventions and cooking pedagogy have reinforced the role of women in cooking (Slater, 2013). Gender differences in cooking permeate down to time spent cooking (Mancino and Neuman, 2007; Taillie, 2018), which is often described as a barrier to enjoying cooking (Jabs and Devine, 2006; Lavelle et al., 2016). Lastly, gender differences in the current pandemic with respect to cooking and food are emerging in the literature (Leddy et al., 2020). These pandemic related disparities may lead to a differential in threats to well-being related to cooking and other family related duties, as women more often deal with “double duty” and the “second shift” at home with meal preparation (McCabe and de Waal Malefyt, 2015).

## Discussion

### Limitations

One limitation of our review is the lack of rigor across existing cooking studies evaluated in this article, including a lack of control groups, the use of small convenience samples, and the use of unstandardized instruments that weaken conclusions about program efficacy. Due to our review’s focus on cooking activity, we reviewed the literature related to studies that explored the specific behavior of cooking or preparing a meal. However, there are related para-activities to cooking, including food provisioning activities like grocery shopping or gardening that may influence well-being. Cooking may also be a vector for emotion evoked by the presence of food (Kaneko et al., 2018). Moreover, cooking is a component of food literacy skills, and at every level of food literacy (functional, interactive, and critical) there is potential to impact well-being (Slater, 2013; Krause et al., 2018).

Despite our focus on cooking through a positive psychology lens, there is the possibility for psychosocial distress, or threats to well-being, that may occur from cooking too much. This is evident in professional cooking settings; however, opportunities for well-being even in these settings may still occur and are being explored in culinary arts education (Murray and Sweeney, 2019). Our perspective is also limited to that of the individual level, and does not take into account community or societal variables relevant to cooking. Structural barriers to cooking are also not fully taken into account despite their obvious importance. Limited access to fruits and vegetables and over access to convenience foods are present in some under resourced neighborhoods leaving way for an overall limited access to healthful cooking ingredients. For example, despite a general increase in cooking frequency in the United States as reported by Taillie (2018), African Americans did not report increases in cooking activity in recent years. Despite this, we believe the application of the PERMA framework dovetails nicely with the food justice approach (Alkon and Agyeman, 2011) by focusing on opportunities for personal achievement and empowerment of community members in the face of aforementioned structural barriers. Finally, any use of well-being outcomes has an individual and cultural limitation. With respect to individual level variation, measurement of confidence with regard to cooking remains problematic as a key indicator of well-being as self-perceived level of confidence may not coincide with objective skill level (Garcia et al., 2016). Well-being is also defined differently in various cultures. For example, in Japanese culture, an indicator of well-being is a sense of life worth living (Sone et al., 2008). This indicator may not fit squarely into the PERMA model. To this point, the role of cooking and, thus, the potential to relate to well-being also has cultural variation. Existing literature on cooking is grounded in a heteronormative perspective and primarily conducted in Western settings suggesting the viewpoint is limited. In summary, interventions following the PERMA model need to consider multiple factors including the built environment, community capacity, and cultural relevance to the community.

As stated by De Certeau (1984) in his book, The Practice of Everyday Life, “The characteristically subtle logic of ordinary activities comes to light only in details.” Currently, within research and within the American default lifestyle, there may be an under appreciation for the positive effects of cooking, perhaps due to its framing as a chore or activity, thus obscuring our understanding of its contribution to well-being. Cooking’s role in well-being may become illuminated through research, interventions, and messaging that put forth its connections to PERMA facets such as positive affect, meaning, and achievement and their associated benefits. Research is needed to identify aspects of cooking that promote psychosocial well-being to change this perspective and, ideally over time, the associated health outcomes related to risk for chronic disease. Additionally, given the complexity of appraising cooking from this viewpoint, and the complexity of studying cooking as a sole phenomenon of human experience and interaction, there is a need for interdisciplinary work to drive future studies. The prevailing reasoning driving policy proposals to date to promote and support cooking are related to its nutritional benefits. Based on this article, it may also be of significance for overall public health to evaluate cooking as a promoter of well-being and flourishing at the individual, household and ultimately societal level. Research in this area is buoyed by trends in increasing interest in cooking on social media and TV programs, suggesting strong public support.

## Data Availability Statement

The original contributions presented in the study are included in the article/supplementary material, further inquiries can be directed to the corresponding author.

## Author Contributions

NF and EC were jointly responsible for manuscript conceptualization, methodology, writing, review, and editing. All authors contributed to the article and approved the submitted version.

### Conflict of Interest

The authors declare that the research was conducted in the absence of any commercial or financial relationships that could be construed as a potential conflict of interest. The statements and contents expressed in this perspective by NF are those of the author and do not reflect the official position of the NIH, DHHS, and/or the United States Government.

## References

[ref1] Adam M. Young-Wolff K. C. Konar E. Winkleby M. (2015). Massive open online nutrition and cooking course for improved eating behaviors and meal composition. Int. J. Behav. Nutr. Phys. Act. 12:143. 10.1186/s12966-015-0305-2, PMID: 26630879 PMC4668707

[ref2] Aiello L. C. Wheeler P. (1995). The expensive-tissue hypothesis: the brain and the digestive system in human and primate evolution. Curr. Anthropol. 36, 199–221. 10.1086/204350

[ref3] Alkon A. H. Agyeman J. (2011). Cultivating food justice: Race, class, and sustainability. Cambridge, MA: MIT press.

[ref4] Ascenso S. Perkins R. Williamon A. (2018). Resounding meaning: a PERMA wellbeing profile of classical musicians. Front. Psychol. 9:1895. 10.3389/fpsyg.2018.01895, PMID: 30459665 PMC6232231

[ref5] Bandura A. (2005). “The evolution of social cognitive theory” in Great minds in management. eds. Smith K. G. Hitt M. A. (Oxford: Oxford University Press), 9–35.

[ref6] Bandura A. (2010). “Self-efficacy” in The Corsini encyclopedia of psychology. eds. Weiner I. B. Craighead W. E., 1–3.

[ref7] Barak-Nahum A. Haim L. B. Ginzburg K. (2016). When life gives you lemons: the effectiveness of culinary group intervention among cancer patients. Soc. Sci. Med. 166, 1–8. 10.1016/j.socscimed.2016.07.046, PMID: 27522112

[ref8] Bartholomaeus J. D. Van Agteren J. E. M. Iasiello M. P. Jarden A. Kelly D. (2019). Positive aging: the impact of a community wellbeing and resilience program. Clin. Gerontol. 42, 377–386. 10.1080/07317115.2018.1561582, PMID: 30654716

[ref9] Berge J. M. Rowley S. Trofholz A. Hanson C. Rueter M. MacLehose R. F.. (2014). Childhood obesity and interpersonal dynamics during family meals. Pediatrics 134, 923–932. 10.1542/peds.2014-1936, PMID: 25311603 PMC4210801

[ref10] Berridge K. C. Kringelbach M. L. (2013). Neuroscience of affect: brain mechanisms of pleasure and displeasure. Curr. Opin. Neurobiol. 23, 294–303. 10.1016/j.conb.2013.01.017, PMID: 23375169 PMC3644539

[ref11] Birnboim N. J. S. (2001). Measuring kitchen performance: what assessment should we choose? Scand. J. Occup. Ther. 8, 193–202. 10.1080/110381201317166559

[ref12] Blanchflower D. G. Oswald A. J. Stewart-Brown S. (2013). Is psychological well-being linked to the consumption of fruit and vegetables? Soc. Indic. Res. 114, 785–801. 10.1007/s11205-012-0173-y

[ref13] Brown K. W. Ryan R. M. (2003). The benefits of being present: mindfulness and its role in psychological well-being. J. Pers. Soc. Psychol. 84, 822–848. 10.1037/0022-3514.84.4.822, PMID: 12703651

[ref14] Bublitz M. G. Peracchio L. A. Andreasen A. R. Kees J. Kidwell B. Miller E. G.. (2013). Promoting positive change: advancing the food well-being paradigm. J. Bus. Res. 66, 1211–1218. 10.1016/j.jbusres.2012.08.014

[ref15] Butler J. Kern M. L. (2016). The PERMA-profiler: a brief multidimensional measure of flourishing. Int. J. Wellbeing 6, 1–48. 10.5502/ijw.v6i3.526

[ref16] Campbell C. (2005). The craft consumer: culture, craft and consumption in a postmodern society. J. Consum. Cult. 5, 23–42. 10.1177/1469540505049843

[ref19] Cohen S. (2004). Social relationships and health. Am. Psychol. 59, 676–684. 10.1037/0003-066X.59.8.676 15554821

[ref20] Cohen S. Doyle W. J. Skoner D. P. Rabin B. S. Gwaltney J. M. (1997). Social ties and susceptibility to the common cold. JAMA 277, 1940–1944. 10.1001/jama.1997.03540480040036, PMID: 9200634

[ref21] Craske M. G. Craig K. D. (1984). Musical performance anxiety: the three-systems model and self-efficacy theory. Behav. Res. Ther. 22, 267–280. 10.1016/0005-7967(84)90007-X, PMID: 6466277

[ref22] Crookes D. M. Shelton R. C. Tehranifar P. Aycinena C. Gaffney A. O. Koch P.. (2016). Social networks and social support for healthy eating among Latina breast cancer survivors: implications for social and behavioral interventions. J. Cancer Surviv. 10, 291–301. 10.1007/s11764-015-0475-6, PMID: 26202538 PMC4724562

[ref23] Csikszentmihalyi M. (1990). Flow: The psychology of optimal experience. New York, NY: Harper & Row.

[ref25] Cuijpers P. Van Straten A. Warmerdam L. (2007). Behavioral activation treatments of depression: a meta-analysis. Clin. Psychol. Rev. 27, 318–326. 10.1016/j.cpr.2006.11.001, PMID: 17184887

[ref26] Cunliffe V. T. (2016). The epigenetic impacts of social stress: how does social adversity become biologically embedded. Epigenomics 8, 1653–1669. 10.2217/epi-2016-0075, PMID: 27869483 PMC5289034

[ref27] Daniels S. Glorieux I. Minnen J. van Tienoven T. P. (2012). More than preparing a meal? Concerning the meanings of home cooking. Appetite 58, 1050–1056. 10.1016/j.appet.2012.02.040, PMID: 22369954

[ref29] De Certeau M. (1984). Arts de faire. Berkeley, CA: University of California Press.

[ref30] Dean M. O’Kane C. Issartel J. McCloat A. Mooney E. Gaul D.. (2021). Guidelines for designing age-appropriate cooking interventions for children: the development of evidence-based cooking skill recommendations for children, using a multidisciplinary approach. Appetite 161:105125. 10.1016/j.appet.2021.105125, PMID: 33482302 PMC8609961

[ref31] Diener E. Ryan K. (2009). Subjective well-being: a general overview. S. Afr. J. Psychol. 39, 391–406. 10.1177/008124630903900402

[ref32] Diener E. Seligman M. E. P. (2004). Beyond money: toward an economy of well-being. Psychol. Sci. Public Interest 5, 1–31. 10.1111/j.0963-7214.2004.00501001.x, PMID: 26158992

[ref33] Dohle S. Rall S. Siegrist M. (2014). I cooked it myself: preparing food increases liking and consumption. Food Qual. Prefer. 33, 14–16. 10.1016/j.foodqual.2013.11.001

[ref129] Duncan M. (2004). “Promoting mental health through occupation” in Transformation through occupation. eds. Watson R. Swartz L. (London: Whurr publishers).

[ref34] Epley N. Schroeder J. (2014). Mistakenly seeking solitude. J. Exp. Psychol. Gen. 143, 1980–1999. 10.1037/a0037323, PMID: 25019381

[ref35] Everson-Rose S. A. Lewis T. T. (2005). Psychosocial factors and cardiovascular diseases. Annu. Rev. Public Health 26, 469–500. 10.1146/annurev.publhealth.26.021304.144542, PMID: 15760298

[ref36] Farmer N. Touchton-Leonard K. Ross A. (2018). Psychosocial benefits of cooking interventions: a systematic review. Health Educ. Behav. 45, 167–180. 10.1177/1090198117736352, PMID: 29121776 PMC5862744

[ref37] Farmer N. Wallen G. R. Yang L. Middleton K. R. Kazmi N. Powell-Wiley T. M. (2019). Household cooking frequency of dinner among non-Hispanic black adults is associated with income and employment, perceived diet quality and varied objective diet quality, HEI (healthy eating index): NHANES analysis 2007–2010. Nutrients 11:2057. 10.3390/nu11092057, PMID: 31480746 PMC6769568

[ref38] Fredrickson B. L. (2001). The role of positive emotions in positive psychology: the broaden-and-build theory of positive emotions. Am. Psychol. 56, 218–226. 10.1037/0003-066X.56.3.218, PMID: 11315248 PMC3122271

[ref39] Fredrickson B. L. Tugade M. M. Waugh C. E. Larkin G. R. (2003). What good are positive emotions in crisis? A prospective study of resilience and emotions following the terrorist attacks on the United States on September 11th, 2001. J. Pers. Soc. Psychol. 84, 365–376. 10.1037/0022-3514.84.2.365, PMID: 12585810 PMC2755263

[ref40] Friedrich B. Mason O. (2018). Applying positive psychology principles to soccer interventions for people with mental health difficulties. Psychology 9, 372–384. 10.4236/psych.2018.93023

[ref41] Gander F. Proyer R. T. Ruch W. (2016). Positive psychology interventions addressing pleasure, engagement, meaning, positive relationships, and accomplishment increase well-being and ameliorate depressive symptoms: a randomized, placebo-controlled online study. Front. Psychol. 7:686. 10.3389/fpsyg.2016.00686, PMID: 27242600 PMC4873493

[ref42] Garcia A. L. Reardon R. McDonald M. Vargas-Garcia E. J. (2016). Community interventions to improve cooking skills and their effects on confidence and eating behaviour. Curr. Nutr. Rep. 5, 315–322. 10.1007/s13668-016-0185-3, PMID: 27882266 PMC5097072

[ref43] Gatley A. Caraher M. Lang T. (2014). A qualitative, cross cultural examination of attitudes and behaviour in relation to cooking habits in France and Britain. Appetite 75, 71–81. 10.1016/j.appet.2013.12.014, PMID: 24370356

[ref44] Goodman F. Disabato D. Kashdan T. Kauffman S. (2017). Measuring well-being: a comparison of subjective well-being and PERMA. J. Posit. Psychol. 13, 321–332. 10.1080/17439760.2017.1388434

[ref45] Grant A. M. Gino F. (2010). A little thanks goes a long way: explaining why gratitude expressions motivate prosocial behavior. J. Pers. Soc. Psychol. 98, 946–955. 10.1037/a0017935, PMID: 20515249

[ref46] Gray K. (2010). Moral transformation: good and evil turn the weak into the mighty. Soc. Psychol. Personal. Sci. 1, 253–258. 10.1177/1948550610367686

[ref47] Greenberg J. Reiner K. Meiran N. (2012). “Mind the trap”: mindfulness practice reduces cognitive rigidity. PLoS One 7:e36206. 10.1371/journal.pone.0036206, PMID: 22615758 PMC3352909

[ref48] Hasan B. Thompson W. G. Almasri J. Wang Z. Lakis S. Prokop L. J.. (2019). The effect of culinary interventions (cooking classes) on dietary intake and behavioral change: a systematic review and evidence map. BMC Nutr. 5:29. 10.1186/s40795-019-0293-8, PMID: 32153942 PMC7050805

[ref49] Herbert J. Flego A. Gibbs L. Waters E. Swinburn B. Reynolds J.. (2014). Wider impacts of a 10-week community cooking skills program-Jamie’s Ministry of Food, Australia. BMC Public Health 14:1161. 10.1186/1471-2458-14-1161, PMID: 25496263 PMC4295497

[ref50] Hersch D. Perdue L. Ambroz T. Boucher J. L. (2014). Peer reviewed: the impact of cooking classes on food-related preferences, attitudes, and behaviors of school-aged children: a systematic review of the evidence, 2003–2014. Prev. Chronic Dis. 11:E193. 10.5888/pcd11.140267, PMID: 25376015 PMC4222785

[ref51] Hidaka B. H. (2012). Depression as a disease of modernity: explanations for increasing prevalence. J. Affect. Disord. 140, 205–214. 10.1016/j.jad.2011.12.036, PMID: 22244375 PMC3330161

[ref52] Hill K. H. O’Brien K. A. Yurt R. W. (2007). Therapeutic efficacy of a therapeutic cooking group from the patients’ perspective. J. Burn Care Res. 28, 324–327. 10.1097/BCR.0B013E318031A24C, PMID: 17351453

[ref53] Holder M. D. (2019). The contribution of food consumption to well-being. Ann. Nutr. Metab. 74, 44–52. 10.1159/000499147, PMID: 31234181

[ref54] Hollywood L. Surgenor D. Reicks M. McGowan L. Lavelle F. Spence M.. (2018). Critical review of behaviour change techniques applied in intervention studies to improve cooking skills and food skills among adults. Crit. Rev. Food Sci. Nutr. 58, 2882–2895. 10.1080/10408398.2017.1344613, PMID: 28678613

[ref55] Holt-Lunstad J. Smith T. B. Layton J. B. (2010). Social relationships and mortality risk: a meta-analytic review. PLoS Med. 7:e1000316. 10.1371/journal.pmed.1000316, PMID: 20668659 PMC2910600

[ref56] Jabs J. Devine C. M. (2006). Time scarcity and food choices: an overview. Appetite 47, 196–204. 10.1016/j.appet.2006.02.014, PMID: 16698116

[ref58] Jyväkorpi S. Pitkälä K. Kautiainen H. Puranen T. Laakkonen M. Suominen M. (2014). Nutrition education and cooking classes improve diet quality, nutrient intake, and psychological well-being of home-dwelling older people—a pilot study. Morb. Mortal. 1, 4–8.

[ref60] Kaneko D. Toet A. Brouwer A. M. Kallen V. van Erp J. (2018). Methods for evaluating emotions evoked by food experiences: a literature review. Front. Psychol. 9:911. 10.3389/fpsyg.2018.00911, PMID: 29937744 PMC6002740

[ref61] Kaplan S. (2001). Meditation, restoration and the management of mental fatigue. Environ. Behav. 33, 480–506. 10.1177/00139160121973106

[ref62] Kessler R. C. Chiu W. T. Demler O. Walters E. E. (2005). Prevalence, severity, and comorbidity of 12-month DSM-IV disorders in the National comorbidity survey replication. Arch. Gen. Psychiatry 62, 617–627. 10.1001/archpsyc.62.6.617, PMID: 15939839 PMC2847357

[ref140] Killingsworth M. A. Gilbert D. T. (2010). A wandering mind is an unhappy mind. Science 330:932. 10.1126/science.1192439 21071660

[ref63] Klerman G. L. Lavori P. W. Rice J. Reich T. Endicott J. Andreasen N. C.. (1985). Birth-cohort trends in rates of major depressive disorder among relatives of patients with affective disorder. Arch. Gen. Psychiatry 42, 689–693. 10.1001/archpsyc.1985.01790300057007, PMID: 4015310

[ref64] Kottke T. E. Stiefel M. Pronk N. P. (2016). “Well-being in all policies”: promoting cross-sectoral collaboration to improve people’s lives. Prev. Chronic Dis. 13:E52. 10.5888/pcd13.160155, PMID: 27079650 PMC4852755

[ref65] Krause C. Sommerhalder K. Beer-Borst S. Abel T. (2018). Just a subtle difference? Findings from a systematic review on definitions of nutrition literacy and food literacy. Health Promot. Int. 33, 378–389. 10.1093/heapro/daw084, PMID: 27803197 PMC6005107

[ref66] Kubovy M. (1999). “On the pleasures of the mind” in Well-being: The foundations of hedonic psychology. eds. Kahneman D. Diener E. Schwarz N. (New York, NY: Russell Sage Foundation), 134–154.

[ref67] Lai J. S. Hiles S. Bisquera A. Hure A. J. McEvoy M. Attia J. (2013). A systematic review and meta-analysis of dietary patterns and depression in community-dwelling adults. Am. J. Clin. Nutr. 99, 181–197. 10.3945/ajcn.113.069880, PMID: 24196402

[ref68] Lambert K. G. (2006). Rising rates of depression in today’s society: consideration of the roles of effort-based rewards and enhanced resilience in day-to-day functioning. Neurosci. Biobehav. Rev. 30, 497–510. 10.1016/j.neubiorev.2005.09.002, PMID: 16253328

[ref69] Lang T. Caraher M. (2001). Is there a culinary skills transition? Data and debate from the UK about changes in cooking culture. J. Home Econ. Inst. Aust. 8, 2–14.

[ref70] Langer E. J. (1992). Matters of mind: mindfulness/mindlessness in perspective. Conscious. Cogn. 1, 289–305. 10.1016/1053-8100(92)90066-J

[ref72] Lavelle F. McGowan L. Spence M. Caraher M. Raats M. M. Hollywood L.. (2016). Barriers and facilitators to cooking from “scratch” using basic or raw ingredients: a qualitative interview study. Appetite 107, 383–391. 10.1016/j.appet.2016.08.115, PMID: 27567551

[ref73] Leddy A. M. Weiser S. D. Palar K. Seligman H. (2020). A conceptual model for understanding the rapid COVID-19-related increase in food insecurity and its impact on health and healthcare. Am. J. Clin. Nutr. 112, 1162–1169. 10.1093/ajcn/nqaa226, PMID: 32766740 PMC7454255

[ref74] Lee J. Krause A. E. Davidson J. W. (2017). The PERMA well-being model and music facilitation practice: preliminary documentation for well-being through music provision in Australian schools. Res. Stud. Music Educ. 39, 73–89. 10.1177/1321103X17703131

[ref75] Levy J. Auld G. (2004). Cooking classes outperform cooking demonstrations for college sophomores. J. Nutr. Educ. Behav. 36, 197–203. 10.1016/S1499-4046(06)60234-0, PMID: 15544728

[ref76] Mancino L. Neuman C. (2007). Who has time to cook? How family resources influence food preparation. United State Department of Agriculture.

[ref77] Marsh L. E. Kanngiesser P. Hood B. (2018). When and how does labour lead to love? The ontogeny and mechanisms of the IKEA effect. Cognition 170, 245–253. 10.1016/j.cognition.2017.10.012, PMID: 29080469

[ref78] Marx W. Moseley G. Berk M. Jacka F. (2017). Nutritional psychiatry: the present state of the evidence. Proc. Nutr. Soc. 76, 427–436. 10.1017/S0029665117002026, PMID: 28942748

[ref79] McCabe M. de Waal Malefyt T. (2015). Creativity and cooking: motherhood, agency and social change in everyday life. J. Consum. Cult. 15, 48–65. 10.1177/1469540513493202

[ref80] Meloni M. (2014). The social brain meets the reactive genome: neuroscience, epigenetics and the new social biology. Front. Hum. Neurosci. 8:309. 10.3389/fnhum.2014.00309, PMID: 24904353 PMC4033168

[ref81] Mills S. Brown H. Wrieden W. White M. Adams J. (2017). Frequency of eating home cooked meals and potential benefits for diet and health: cross-sectional analysis of a population-based cohort study. Int. J. Behav. Nutr. Phys. Act. 14:109. 10.1186/s12966-017-0567-y, PMID: 28818089 PMC5561571

[ref82] Mills S. Wolfson J. A. Wrieden W. L. Brown H. White M. Adams J. (2020). Perceptions of “Home Cooking”: a qualitative analysis from the United Kingdom and United States. Nutrients 12:198. 10.3390/nu12010198, PMID: 31940897 PMC7019500

[ref83] Mogilner C. Chance Z. Norton M. I. (2012). Giving time gives you time. Psychol. Sci. 23, 1233–1238. 10.1177/0956797612442551, PMID: 22972905

[ref84] Mogilner C. Whillans A. Norton M. I. (2018). “Time, money, and subjective well- being” in Handbook of well-being. Noba scholar handbook series: Subjective well-being. eds. Diener E. Oishi S. Tay L. (Salt Lake City, UT: DEF publishers).

[ref85] Moisio R. Arnould E. J. Price L. L. (2004). Between mothers and markets: constructing family identity through homemade food. J. Consum. Cult. 4, 361–384. 10.1177/1469540504046523

[ref86] Monsivais P. Aggarwal A. Drewnowski A. (2014). Time spent on home food preparation and indicators of healthy eating. Am. J. Prev. Med. 47, 796–802. 10.1016/j.amepre.2014.07.033, PMID: 25245799 PMC4254327

[ref87] Moynihan J. A. Larson M. R. Treanor J. Duberstein P. R. Power A. Shore B.. (2004). Psychosocial factors and the response to influenza vaccination in older adults. Psychosom. Med. 66, 950–953. 10.1097/01.psy.0000140001.49208.2d, PMID: 15564363

[ref88] Mueser K. T. Deavers F. Penn D. L. Cassisi J. E. (2013). Psychosocial treatments for schizophrenia. Annu. Rev. Clin. Psychol. 9, 465–497. 10.1146/annurev-clinpsy-050212-185620, PMID: 23330939

[ref89] Mujcic R. Oswald A. (2016). Evolution of well-being and happiness after increases in consumption of fruit and vegetables. Am. J. Public Health 106, 1504–1510. 10.2105/AJPH.2016.303260, PMID: 27400354 PMC4940663

[ref90] Murphy B. Benson T. McCloat A. Mooney E. Elliott C. Dean M.. (2021). Changes in consumers’ food practices during the COVID-19 lockdown, implications for diet quality and the food system: a cross-continental comparison. Nutrients 13:20. 10.3390/nu13010020, PMID: 33374619 PMC7822477

[ref91] Murray D. Sweeney A. (2019). “The Mindful Kitchen” - The Potential of New Skills to Innovate Kitchen Culture and Futureproof The Chef Career! Available at: https://www.chefnetwork.ie/blogs/annette-sweeney/2019/08/13/the-mindful-kitchen (Accessed March 31, 2020).

[ref92] Mutrie N. Faulkner G. (2004). “Physical activity: positive psychology in motion” in Positive psychology in practice. eds. Linley P. A. Joseph S. (Hoboken, NJ: John Wiley & Sons, Inc.), 146–164.

[ref93] O’neil A. Quirk S. E. Housden S. Brennan S. L. Williams L. J. Pasco J. A.. (2014). Relationship between diet and mental health in children and adolescents: a systematic review. Am. J. Public Health 104, e31–e42. 10.2105/AJPH.2014.302110, PMID: 25208008 PMC4167107

[ref94] Opie R. S. O’Neil A. Itsiopoulos C. Jacka F. N. (2015). The impact of whole-of-diet interventions on depression and anxiety: a systematic review of randomised controlled trials. Public Health Nutr. 18, 2074–2093. 10.1017/S1368980014002614, PMID: 25465596 PMC10271872

[ref95] Raber M. Patterson M. Jia W. Sun M. Baranowski T. (2018). Utility of eButton images for identifying food preparation behaviors and meal-related tasks in adolescents. Nutr. J. 17:32. 10.1186/s12937-018-0341-2, PMID: 29477143 PMC6389239

[ref96] Reicks M. Kocher M. Reeder J. (2018). Impact of cooking and home food preparation interventions among adults: a systematic review (2011–2016). J. Nutr. Educ. Behav. 50, 148–172. 10.1016/j.jneb.2017.08.004, PMID: 28958671

[ref97] Reicks M. Trofholz A. C. Stang J. S. Laska M. N. (2014). Impact of cooking and home food preparation interventions among adults: outcomes and implications for future programs. J. Nutr. Educ. Behav. 46, 259–276. 10.1016/j.jneb.2014.02.001, PMID: 24703245 PMC4063875

[ref98] Robins L. N. Helzer J. E. Weissman M. M. Orvaschel H. Gruenberg E. Burke J. D.. (1984). Lifetime prevalence of specific psychiatric disorders in three sites. Arch. Gen. Psychiatry 41, 949–958. 10.1001/archpsyc.1984.01790210031005, PMID: 6332590

[ref99] Rombauer I. S. Becker M. R. Becker E. Becker J. Scott M. (2019). Joy of cooking: 2019 edition fully revised and updated. New York, NY: Scribner.

[ref100] Ryff C. D. Singer B. (2001). Emotion, social relationships, and health. USA: Oxford University Press.

[ref101] Saad L. (2017). Eight in 10 Americans afflicted by stress. Washington, DC, USA: Gallup.

[ref102] Seligman M. (2010). “Flourish: positive psychology and positive interventions” in The Tanner lectures on human values. Vol. 31. 1–56.

[ref103] Seligman M. E. (2011). Flourish: A visionary new understanding of happiness and well-being. New York, NY: Simon and Schuster.

[ref104] Seligman M. (2018). PERMA and the building blocks of well-being. J. Posit. Psychol. 13, 333–335. 10.1080/17439760.2018.1437466

[ref105] Seligman M. Csikszentmihalyi M. (2000). Positive psychology: an introduction. Am. Psychol. 55, 5–14. 10.1037/0003-066X.55.1.5, PMID: 11392865

[ref106] Sells D. J. Stayner D. A. Davidson L. (2004). Recovering the self in schizophrenia: an integrative review of qualitative studies. Psychiatry Q. 75, 87–97. 10.1023/B:PSAQ.0000007563.17236.97, PMID: 14992305

[ref107] Short F. (2003). Domestic cooking skills-what are they. J. HEIA 10, 13–22.

[ref108] Short F. (2006). Kitchen secrets: The meaning of cooking in everyday life. Oxford, England: Berg.

[ref109] Short F. (2007). “Cooking; convenience and dis-connection” in *Inter: A European Cultural Studies: Conference in Sweden 11–13 June 2007*; Linköping University Electronic Press, 553–563.

[ref110] Sidenvall B. Nydahl M. Fjellström C. (2000). The meal as a gift—the meaning of cooking among retired women. J. Appl. Gerontol. 19, 405–423. 10.1177/073346480001900403

[ref111] Simmons D. Chapman G. E. (2012). The significance of home cooking within families. Br. Food J. 114, 1184–1195. 10.1108/00070701211252110

[ref112] Sin N. L. Lyubomirsky S. (2009). Enhancing well-being and alleviating depressive symptoms with positive psychology interventions: a practice-friendly meta-analysis. J. Clin. Psychol. 65, 467–487. 10.1002/jclp.20593, PMID: 19301241

[ref113] Slater J. (2013). Is cooking dead? The state of home economics food and nutrition education in a Canadian province. Int. J. Consum. Stud. 37, 617–624. 10.1111/ijcs.12042

[ref114] Slater P. J. Edwards R. M. Badat A. A. (2018). Evaluation of a staff well-being program in a pediatric oncology, hematology, and palliative care services group. J. Healthc. Leadersh. 10, 67–85. 10.2147/JHL.S176848, PMID: 30532609 PMC6241860

[ref115] Smith K. J. McNaughton S. A. Gall S. L. Blizzard L. Dwyer T. Venn A. J. (2010). Involvement of young Australian adults in meal preparation: cross-sectional associations with sociodemographic factors and diet quality. J. Am. Diet. Assoc. 110, 1363–1367. 10.1016/j.jada.2010.06.011, PMID: 20800130

[ref116] Soga M. Gaston K. J. Yamaura Y. (2016). Gardening is beneficial for health: a meta-analysis. Prev. Med. Rep. 5, 92–99. 10.1016/j.pmedr.2016.11.007, PMID: 27981022 PMC5153451

[ref117] Soliah L. A. L. Walter J. M. Jones S. A. (2012). Benefits and barriers to healthful eating: what are the consequences of decreased food preparation ability? Am. J. Lifestyle Med. 6, 152–158. 10.1177/1559827611426394

[ref118] Sone T. Nakaya N. Ohmori K. Shimazu T. Higashiguchi M. Kakizaki M.. (2008). Sense of life worth living (ikigai) and mortality in Japan: Ohsaki study. Psychosom. Med. 70, 709–715. 10.1097/PSY.0b013e31817e7e64, PMID: 18596247

[ref119] Steptoe A. Wardle J. Marmot M. (2005). Positive affect and health-related neuroendocrine, cardiovascular, and inflammatory processes. Proc. Natl. Acad. Sci. U. S. A. 102, 6508–6512. 10.1073/pnas.0409174102, PMID: 15840727 PMC1088362

[ref120] Stone A. A. Smyth J. M. Pickering T. Schwartz J. (1996). Daily mood variability: form of diurnal patterns and determinants of diurnal patterns. J. Appl. Soc. Psychol. 26, 1286–1305. 10.1111/j.1559-1816.1996.tb01781.x

[ref121] Taillie L. S. (2018). Who’s cooking? Trends in US home food preparation by gender, education, and race/ethnicity from 2003 to 2016. Nutr. J. 17:41. 10.1186/s12937-018-0347-9, PMID: 29609591 PMC5881182

[ref122] Tugade M. M. Fredrickson B. L. (2007). Regulation of positive emotions: emotion regulation strategies that promote resilience. J. Happiness Stud. 8, 311–333. 10.1007/s10902-006-9015-4

[ref123] Twenge J. M. Cooper A. B. Joiner T. E. Duffy M. E. Binau S. G. (2019). Age, period, and cohort trends in mood disorder indicators and suicide-related outcomes in a nationally representative dataset, 2005–2017. J. Abnorm. Psychol. 128, 185–119. 10.1037/abn0000410, PMID: 30869927

[ref124] Uchino B. N. Uno D. Holt-Lunstad J. (1999). Social support, physiological processes, and health. Curr. Dir. Psychol. Sci. 8, 145–148.

[ref125] Utter J. Denny S. Lucassen M. Dyson B. (2016). Adolescent cooking abilities and behaviors: associations with nutrition and emotional well-being. J. Nutr. Educ. Behav. 48, 35–41. 10.1016/j.jneb.2015.08.016, PMID: 26411900

[ref127] VanderWeele T. J. (2017). On the promotion of human flourishing. Proc. Natl. Acad. Sci. U. S. A. 114, 8148–8156. 10.1073/pnas.1702996114, PMID: 28705870 PMC5547610

[ref128] Wadley L. Backwell L. d’Errico F. Sievers C. (2020). Cooked starchy rhizomes in Africa 170 thousand years ago. Science 367, 87–91. 10.1126/science.aaz5926, PMID: 31896717

[ref130] Whang W. Kubzansky L. D. Kawachi I. Rexrode K. M. Kroenke C. H. Glynn R. J.. (2009). Depression and risk of sudden cardiac death and coronary heart disease in women: results from the Nurses’ Health Study. J. Am. Coll. Cardiol. 53, 950–958. 10.1016/j.jacc.2008.10.060, PMID: 19281925 PMC2664253

[ref131] Whillans A. V. Dunn E. W. Smeets P. Bekkers R. Norton M. I. (2017). Buying time promotes happiness. Proc. Natl. Acad. Sci. U. S. A. 114, 8523–8527. 10.1073/pnas.1706541114, PMID: 28739889 PMC5559044

[ref132] Wolfson J. A. Bleich S. N. (2015). Is cooking at home associated with better diet quality or weight-loss intention? Public Health Nutr. 18, 1397–1406. 10.1017/S1368980014001943, PMID: 25399031 PMC8728746

[ref133] Wolfson J. A. Bleich S. N. Smith K. C. Frattaroli S. (2016). What does cooking mean to you?: perceptions of cooking and factors related to cooking behavior. Appetite 97, 146–154. 10.1016/j.appet.2015.11.030, PMID: 26654888

[ref134] Wolfson J. A. Bostic S. Lahne J. Morgan C. Henley S. C. Harvey J.. (2017). A comprehensive approach to understanding cooking behavior. Br. Food J. 119, 1147–1158. 10.1108/BFJ-09-2016-0438

[ref135] World Health Organization (2013). Global action plan for the prevention and control of noncommunicable diseases 2013–2020. Geneva, Switzerland: World Health Organization.

[ref136] Worsley A. Wang W. Ismail S. Ridley S. (2014). Consumers’ interest in learning about cooking: the influence of age, gender and education. Int. J. Consum. Stud. 38, 258–264. 10.1111/ijcs.12089

[ref137] Wrangham R. W. Jones J. H. Laden G. Pilbeam D. Conklin-Brittain N. Brace C. L.. (1999). The raw and the stolen: cooking and the ecology of human origins. Curr. Anthropol. 40, 567–594. 10.1086/300083, PMID: 10539941

